# Environmental Contamination Prevalence, Antimicrobial Resistance and Molecular Characteristics of Methicillin-Resistant *Staphylococcus Aureus* and *Staphylococcus Epidermidis* Isolated from Secondary Schools in Guangzhou, China

**DOI:** 10.3390/ijerph17020623

**Published:** 2020-01-18

**Authors:** Yingying Wang, Jialing Lin, Ting Zhang, Suiping He, Ying Li, Wencui Zhang, Xiaohua Ye, Zhenjiang Yao

**Affiliations:** 1 Department of Epidemiology and Health Statistics, Guangdong Pharmaceutical University, Guangzhou 510310, China; wyying425@126.com (Y.W.); 18826238076@163.com (S.H.); lyshine691@163.com (Y.L.); zhangwencui0704@163.com (W.Z.); 2 School of Public Health and Community Medicine, The University of New South Wales, Sydney 2052, Australia; jialing.lin@student.unsw.edu.au; 3 Department of immunization programme, Dongguan Center for Disease Control and Prevention, Dongguan 523000, China; tzhang92@163.com

**Keywords:** Methicillin-resistant *Staphylococcus aureus*, Methicillin-resistant *Staphylococcus epidermidis*, school environment, contamination, antimicrobial resistance, molecular characteristics

## Abstract

*Background*: Methicillin-resistant *Staphylococcus aureus* (MRSA) and *Staphylococcus epidermidis* (MRSE), the most prevalent causes of hospital-associated and community-associated infections, could exist on frequently touched surfaces. This study aims to determine the contamination prevalence and the characteristics of MRSA and MRSE isolated from secondary school environments. *Methods*: We collected environmental samples from ten secondary schools in Guangzhou city between October 2016 and January 2017. The samples were confirmed for MRSA and MRSE isolates by using biochemical tests and polymerase chain reactions. Antimicrobial susceptibility testing was performed by the Kirby-Bauer disk diffusion method. Staphylococcal cassette chromosome mec (SCC*mec*) typing, toxin gene screening, and multilocus sequence typing (MLST) were performed to further characterize the isolates. Data were analyzed by two-sample proportion tests. *Results*: A total of 1830 environmental samples were collected. The prevalence of MRSA and MRSE contamination were 1.86% (34/1830) and 5.14% (94/1830), respectively. The proportions of multidrug resistance in both MRSA (58.82%) and MRSE (63.83%) isolates were high. Seven clonal complexes (CC) and 12 sequence types (ST) were identified, with the CC5 (35.29%) and ST45 (25.53%) being the most prevalent. We found that 44.12% of the MRSA isolates were community-acquired and the main type was ST45-SCC*mec* IV. We found that 5.88% and 32.35% of MRSA isolates were positive to Panton-Valentine leukocidin (PVL) and toxic shock syndrome toxin-1 (*tst*) gene, respectively. No MRSE isolate was positive to the toxin genes. *Conclusion*: Our findings raise potential public health concerns for environmental contamination of MRSA and MRSE in school environments. Surfaces of school environments may potentially provide a source for cross-contamination with these bacteria into the wider community.

## 1. Introduction

Methicillin-resistant *Staphylococcus aureus* (MRSA) is the leading cause of hospital-associated (HA) and community-associated (CA) infections ranging from minor skin and soft tissue infections to life-threatening pneumonia [1,2]. MRSA infections, particularly infections caused by multidrug-resistant (MDR) *Staphylococcus aureus*, are becoming more and more common in clinical facilities [3]. In addition, *Staphylococcus epidermidis*, a normal inhabitant of human skin and mucous membranes, has become a significant conditional pathogen in the hospital environment with resistance to antibiotics such as methicillin-resistant *Staphylococcus epidermidis* (MRSE) [4,5]. Methicillin-resistant isolates are resistant to most β-lactam antibiotics and multiple antibiotics, which can increase the mortality and health care costs of staphylococcal infections [6].

Numerous studies have shown the role of the environment as a reservoir for pathogens, and proved that environmental contamination with an infection strain is a significant and independent risk factor in transmission [7,8,9]. There was increasing cross-over transmission with the blurring of the boundaries between HA- and CA-*Staphylococci* [10]. Methicillin-resistant *Staphylococci* (including MRSA and MRSE) have been found in communities, such as public transports [8,11], parks [12], beaches [13], and universities [14]. However, data on the epidemiology of methicillin-resistant *Staphylococci* in school environment is limited in China. Therefore, this study aims to elucidate the contamination prevalence, antimicrobial resistance, and molecular characteristics of methicillin-resistant *Staphylococci* in school environments in Guangzhou, China.

## 2. Methods

### 2.1. Study Design and Setting

This cross-sectional study was conducted in ten secondary schools in Guangzhou, China from October 2016 to January 2017. A stratified sampling process was conducted to choose secondary schools. First, all the secondary schools were divided into urban and rural categories. Then, five schools were selected from the urban region and five from the rural region using simple random sampling. The head teachers of the selected schools signed an informed consent form.

### 2.2. Environmental Locations and Sampling

The following places and locations/objects from each school were selected—classrooms (doorknobs, switches, desks, and chairs), toilets (doorknobs, switches, flush handles, and faucet handles), stairs (handrails), corridors (handrails), and playgrounds (balls, horizontal bars, and chairs). These locations were selected because they are frequently touched by students and teachers, and could easily be a reservoir for the organisms. In each school, we collected 183 samples, including 84 from classrooms, 54 from toilets, 15 from stairs, 15 from corridors, and 15 from playgrounds. A total of 1830 environmental samples were collected from these ten schools.

Each environmental location/object was classified and numbered by the investigators before sampling. We also recorded the weather during sampling (including sunny, cloudy, and rainy day). Sterile cotton swabs moistened with 0.9% saline water were used to wipe surfaces of environmental objects. Then each swab was put into a sterile tube with 7.5% sodium chloride broth and transferred to the laboratory within 4 h for further experiments.

### 2.3. Isolation and Identification of Bacterial Strains

Swabs were incubated at 37 ± 1 °C for 24 h and then transferred to mannitol salt agar (each liter containing 10g petone, 1g beef extract, 10g mannitol, 75g sodium chloride, 0.025g phenol red, and 14g agar) with inoculating loop for another 24h of incubation. The colonies which were grape-like clusters and gram-positive under the microscope were further screened for the catalase reaction, *β*-hemolysin, and tube coagulase test. Isolates were identified as *Staphylococcus aureus* (*S. aureus*) if they were positive for all the aforementioned tests as well as positive for the *16S rRNA* and *nuc* genes [15]. *Staphylococcus epidermidis* (*S. epidermidis*) isolates were confirmed if they were negative for the tube coagulase test as well as positive for the *16S rRNA* and *epi* genes [16]. The *S. aureus* and *S. epidermidis* isolates that were positive for the *mecA* gene and/or resistant to cefoxitin were identified as MRSA and MRSE, respectively.

### 2.4. Antimicrobial Susceptibility Testing

Antimicrobial susceptibility testing was conducted by the Kirby-Bauer disk diffusion method based on the Clinical and Laboratory Standards Institute guidelines, 2016 [17]. The following 12 antimicrobial agents were tested—penicillin, cefoxitin, erythromycin, trimethoprim-sulfamethoxazole, rifampicin, clindamycin, tetracycline, teicoplanin, chloramphenicol, gentamicin, moxifloxacin, and linezolid. The isolates that were resistant to ≥1 agent in ≥3 antimicrobial categories were classified as multidrug resistant (MDR) [18]. *S. aureus* ATCC25923 and *S. epidermidis* ATCC12228 (American Type Culture Collection) were used for the quality controls.

### 2.5. Molecular Characteristic

All the *S. aureus* and *S. epidermidis* isolates were tested for the presence of toxin genes including toxic shock syndrome toxin-1 gene (*tst*), staphylococcal enterotoxins (*sea*, *seb*), and haemolysin gene (*hla*) by using multiplex polymerase chain (PCR) assay [19,20]. The *S. aureus* isolates were also tested for the Panton-Valentine leukocidin (PVL) genes [15] and the multilocus sequence typing (MLST) [21]. All the MRSA isolates were tested for the staphylococcal cassette chromosome *mec* (SCC*mec*) typing [22]. The nucleotide sequences of the primers and the size of the PCR products of genes can be found in Table S1. More details are available in our previous publication [23].

### 2.6. Statistical Analysis

The data were entered into a computerized database using Epidata 3.1 (EpiData Association, Odense, Denmark) and exported to Stata 15.0 (College Station, TX, USA) software for further statistical analysis. The significant difference between proportions of categorical variables was analyzed by using Pearson’s chi-squared test or Fisher’s exact test for small samples. Odds ratios (ORs) with 95% confidence interval (CI) were used to assess the antibiotic resistance risk between MRSA and MRSE isolates. The *p* value < 0.05 (two-sided) was considered statistically significant.

## 3. Results

### 3.1. Prevalence of MRSA and MRSE Contamination

The distribution of MRSA and MRSE isolates is shown in Table 1. The prevalence of MRSA and MRSE contamination were 1.86% (34/1830) and 5.14% (94/1830), respectively. The prevalence of MRSA contamination among different places were significantly different (*χ*^2^ = 12.84, *p* = 0.012). The highest prevalence of MRSA contamination was the classroom (3.10%), followed by the corridor, stair, and toilet, while no MRSA isolate was detected from the playground. There was no significant difference in the prevalence of MRSE contamination among different places or objects (*p* > 0.05). We also found that weather and area had no effect on the prevalence of MRSA (weather: *χ*^2^ = 2.220, *p* = 0.136; area: *χ*^2^ = 0.043, *p* = 0.836) or MRSE (weather: *χ*^2^ = 0.042, *p* = 0.837; area: *χ*^2^ = 0.035, *p* = 0.853) contamination.

### 3.2. Antimicrobial Susceptibility Profiles

**Figure 1:**
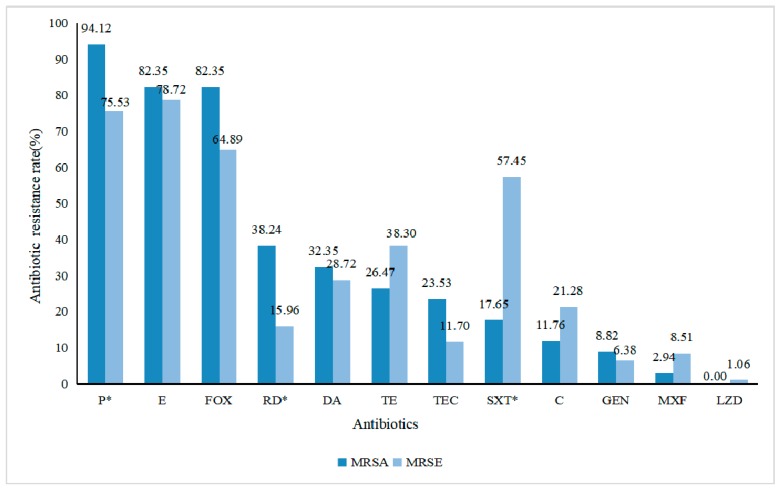
Antibiotic resistance rate of MRSA and MRSE isolates. Note: * There was a statistically significant difference, *p* < 0.05.

**Figure 2:**
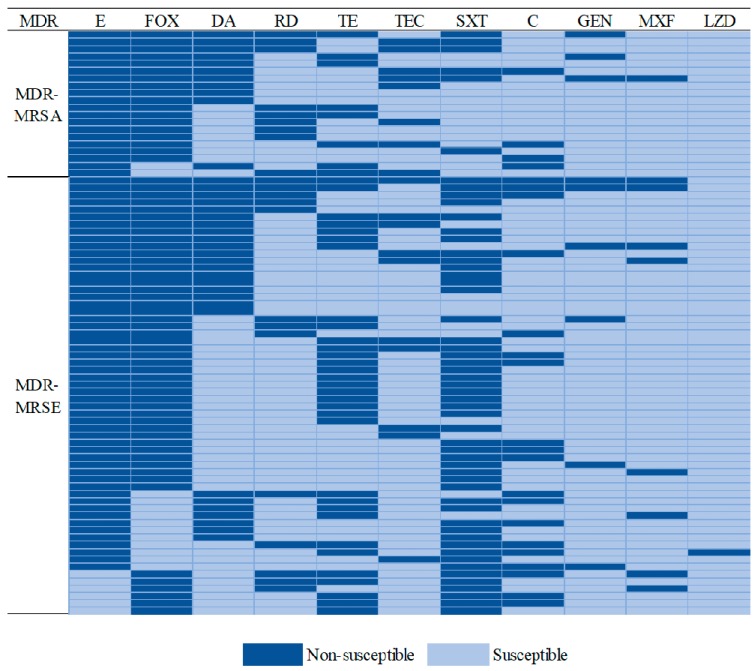
Proportions of antibiotic resistance between MDR-MRSA and MDR-MRSE isolates.

The proportions of antimicrobial resistance are shown in Figure 1. Both MRSA and MRSE isolates were with high proportions of resistance to penicillin, erythromycin, and cefoxitin. We found that the MRSA isolates were more likely to be resistant to penicillin (OR, 5.18; 95% CI, 1.15–23.32; *p =* 0.022) and rifampin (OR, 3.26; 95% CI, 1.35–7.90; *p =* 0.007) than the MRSE isolates. The MRSA isolates were less likely to be resistant to trimethoprim-sulfamethoxazole (OR, 0.16; 95% CI, 0.06–0.42; *p* < 0.001) than the MRSA isolates. Notably, 58.82% of the MRSA and 63.83% of the MRSE isolates were MDR. The predominant MDR pattern of the MRSA isolates was co-resistance to cefoxitin, erythromycin, and clindamycin, while the pattern of the MRSE isolates was co-resistance to erythromycin, cefoxitin, and trimethoprim-sulfamethoxazole (Figure 2). There was no significant difference in antibiotic resistance between the MDR-MRSA and MDR-MRSE isolates (OR, 0.81; 95% CI, 0.34–1.97; *p =* 0.61).

### 3.3. Molecular Characteristics

**Figure 3:**
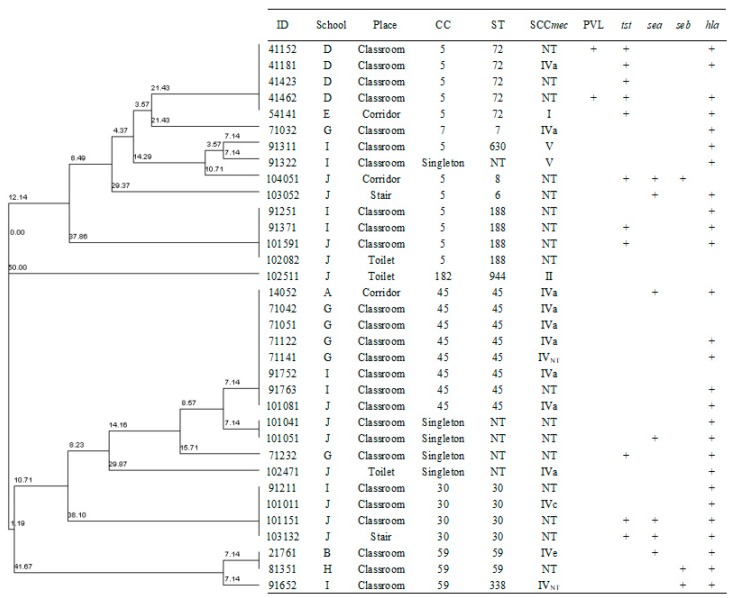
Clonal dendrogram and detailed information of MRSA isolates.

**Figure 4:**
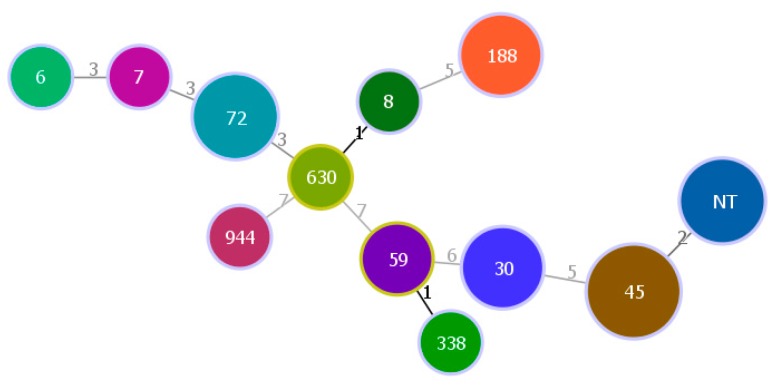
Minimum spanning tree of sequence types of MRSA isolates.

Six clonal complexes (CCs) and 11 sequence types (STs) were found in the MRSA isolates. The most predominant CC was CC5 (35.29%, 12/34), followed by CC45 (23.53%, 8/34), CC30 (11.76%, 4/34), CC59 (8.82%, 3/34), and CC182 (2.94%, 1/34). Notably, the MRSA isolates with the same CC were from different places and different schools. From Figure 3 and Figure 4, we found that the three most predominant STs were ST45 (23.53%, 8/34), ST72 (14.71%, 5/34), and ST188 (11.76%, 4/34).

As to virulence genes, only two CC5 MRSA isolates were positive for the PVL genes. We found that 32.35% of the MRSA isolates (11/34) were positive for the *tst* gene, and most of these 11 isolates were CC5 (23.53%, 8/34). Seven (20.59%) MRSA isolates were positive for the *sea* gene, 3 (8.82%) for the *seb* gene, and 27 (79.41%) for the *hla* gene. All the MRSE isolates were negative to the PVL, *tst, sea*, *seb,* and *hla* genes.

Four SCC*mec* types were observed in 34 MRSA isolates (Figure 3), including type IV (38.24%, 13/34), V (5.88%, 2/34), II (2.94%, 1/34), and I (2.94%, 1/34), while 17 isolates (50.00%) were non-typeable (NT).

## 4. Discussion

To the best of our knowledge, this study contributes to the contamination prevalence, phenotypic and molecular characteristics of methicillin-resistant *Staphylococci* in the school environment in China. A total of 34 MRSA and 94 MRSE isolates were found from ten secondary schools in Guangzhou, China. The proportions of MDR in both MRSA and MRSE isolates were high. Diverse CCs and STs were found in the MRSA isolates. Most MRSA isolates were CA-MRSA and the main type was ST45-IV.

The prevalence of MRSA (1.86%) contamination in this study was lower than studies conducted in high schools [24], universities [25], and public transports [8,26,27], but was higher than a study conducted in secondary schools in Canada (0.68%) [28]. The prevalence of MRSE (5.14%) contamination was lower than observed studies [14,29]. Differences in sampling methods, sampling techniques, and geographical locations could partially explain the variation of prevalence. Moreover, the prevalence of MRSA contamination in classrooms (3.10%) in this study was higher than observed studies [25,28]. This might be due to these areas being more frequently touched in classrooms than in other places.

The patterns of antibiotic resistance in both MRSA and MRSE isolates were similar to observed studies [8,29], which also found high proportions of antibiotic resistance to penicillin, erythromycin, and cefoxitin. We found higher proportions of MDR in both MRSA (58.82%) and MRSE (63.83%) isolates in this study than previous studies [2,5,9], which might be due to the overuse of antibiotics by school students.

The results of molecular characteristics further broadened our insights into the epidemiology of methicillin-resistant *Staphylococci* in a non-hospital environment. The main SCC*mec* types of MRSA isolates were IV and V, which shows most of them were CA-MRSA [27]. The results of the MLST show that ST45 was the most predominant type of MRSA isolates, similar to a previously published school environment study [30]. However, the most predominant CC type of MRSA isolates was CC5, which was reported to be one of the main CC types for HA-MRSA [31,32]. Thus, the results of SCC*mec* and MLST could indicate there was cross-transmission between the hospitals and communities. Additionally, some MRSA isolates from different schools/places/locations displayed identical molecular characteristics, also suggesting there was cross-transmission between the different places or/and schools.

We found that 5.88% of the MRSA isolates were positive for PVL genes, which was higher than public transports [8]. We also found a higher proportion of *tst* gene (23.53%) in CC5 MRSA isolates than a hospital study (17.3%) [31]. No MRSE isolate was positive for toxin genes. These findings help demonstrate that the MRSA isolates are more virulent than MRSE isolates.

Although this study contributes to the epidemiology of MRSA and MRSE contamination in the school environment in China, there are still some limitations. Firstly, we could not evaluate the dynamic change of isolates because of the cross-sectional design. Secondly, we could not elucidate the association between the school environment and students because we did not concurrently collect samples from the students, but we will consider it in future studies. Thirdly, due to the financial limitation we did not explore many more molecular characteristics for the MRSE isolates, which to some extent would lead to a limitation in elucidating the association between the MRSE and MRSA isolates.

## 5. Conclusions

In conclusion, our findings raise potential public health concerns for the environmental contamination of MRSA and MRSE in the school environment. Surfaces of the school environment may potentially provide a source for cross-contamination with these bacteria into the wider community. Effective disinfection measures should be taken in the school environment so as to decrease and prevent methicillin-resistant *Staphylococci* contamination and transmission.

## Figures and Tables

**Table 1:** Distribution of places and objects on MRSA and MRSE isolates in secondary schools in Guangzhou, China.

Location	N	MRSA			MRSE		
		*n* (%)	*χ* ^2^	*p* value	*n* (%)	*χ* ^2^	*p* value
Places	1830	34 (1.86)	12.84	0.012	94 (5.14)	2.68	0.612
Classroom	840	26 (3.10)			52 (6.19)		
Toilet	540	3 (0.56)			16 (2.96)		
Stair	150	2 (1.33)			10 (6.67)		
Corridor	150	3 (2.00)			11 (7.33)		
Playground	150	0 (0.00)			5 (3.33)		
Objects	1830	34 (1.86)	11.48	0.244	94 (5.14)	4.48	0.877
Doorknob	300	4 (1.33)			12 (4.00)		
Light switch	120	3 (2.50)			2 (1.67)		
Electric switch	60	5 (8.33)			3 (5.00)		
Desk	300	11 (3.67)			20 (6.67)		
Chair	320	4 (1.25)			24 (7.50)		
Toilet flush handle	180	1 (0.56)			5 (2.78)		
Faucet handle	120	1 (0.83)			4 (3.33)		
Handrail	300	5 (1.67)			21 (7.00)		
Horizontal bar	50	0 (0.00)			1 (2.00)		
Ball	80	0 (0.00)			2 (2.50)		

## Supplementary Materials

The following are available online at https://www.mdpi.com/1660-4601/17/2/623/s1, Table S1. Nucleotide sequences of primers and size of PCR products (bp) of genes.

## Abbreviations

P: penicillin; E: erythromycin; FOX: cefoxitin; RD: rifampicin; DA: clindamycin; TE: tetracycline; TEC: teicoplanin; SXT: trimethoprim-sulfamethoxazole; C: chloramphenicol; GEN: gentamicin; MXF: moxifloxacin; LZD: linezolid; MDR: multidrug resistant.
