# Supplementary material for: Environmental Contamination Prevalence, Antimicrobial Resistance and Molecular Characteristics of Methicillin-Resistant Staphylococcus Aureus and Staphylococcus Epidermidis Isolated from Secondary Schools in Guangzhou, China
Source: Int J Environ Res Public Health. 2020 Jan 18;17(2):623. doi: 10.3390/ijerph17020623 (PMC7013935; doi:10.3390/ijerph17020623)
Supplement: Supplementary file 1 [file ijerph-17-00623-s001.pdf]

Supplementary Table S1. Nucleotide sequences of primers and size of PCR products (bp) of genes.

Gene	Primer	Oligonucleotides sequence (5′–3′)	PCR product (bp)
16SrRNA	16SrRNA-1	AACTCTGTTATTAGGGAAGAACA	756
	16SrRNA-2	CCACCTTCCTCCGGTTTGTCACC	
nuc	nuc-1	GCGATTGATGGTGATACGGTT	279
	muc-2	AGCCAAGCCTTGACGAACTAAAGC	
epi	epi-1	TTGTAAACCATTCTGGACCG	251
	epi-2	ATGCGTGAGATACTTCTTCG	
mecA	mecA-1	AAAATCGATGGTAAAGGTTGGC	533
	mecA-2	AGTTCTGCAGTACCGGATTTGC	
PVL	pvl-1	ATCATTAGGTAAAATGTCTGGACATGATCCA	433
	pvl-2	GCATCAAGTGTATTGGATAGCAAAAGC	
tst	tst-1	ACCCCTGTTCCCTTATCATC	326
	tst-2	TTTTCAGTATTTGTAACGCC	
sea	sea-1	GGTTATCAATGTGCGGGTGG	102
	sea-2	CGGCACTTTTTTCTCTTCGG	
seb	seb-1	GTATGGTGGTGTAACTGAGC	164
	seb-2	CCAAATAGTGACGAGTTAGG	
hla	hla-1	CTGATTACTATCCAAGAAATTCGATTG	209
	hla-2	CTTTCCAGCCTACTTTTTTATCAGT	
SCC*mec* I	Type I-1	GCTTTAAAGAGTGTCGTTACAGG	613
	Type I-2	GTTCTCTCATAGTATGACGTCC	
SCC*mec* II	Type II-1	CGTTGAAGATGATGAAGCG	398
	Type II-2	CGAAATCAATGGTTAATGGACC	
SCC*mec* III	Type III-1	CCATATTGTGTACGATGCG	280
	Type III-2	CCTTAGTTGTCGTAACAGATCG	
SCC*mec* IVa	Type IVa -1	GCCTTATTCGAAGAAACCG	776
	Type IVa -2	CTACTCTTCTGAAAAGCGTCG	
SCC*mec* IVb	Type IVc -1	TCTGGAATTACTTCAGCTGC	493
	Type IVc -2	AAACAATATTGCTCTCCCTC	
SCC*mec* IVc	Type IVc -1	CCTGAATCTAAAGAGATACACCG	200
	Type IVc -2	GGTTATTTTCATAGTGAATCGC	
SCC*mec* IVd	Type IVd -1	CTCAAAATACGGACCCCAATACA	881
	Type IVd -2	TGCTCCAGTAATTGCTAAAG	
SCC*mec* V	Type V-1	GAACATTGTTACTTAAATGAGCG	325
	Type V-2	TGAAAGTTGTACCCTTGACACC	
